# CLIPing the brain: Studies of protein–RNA interactions important for neurodegenerative disorders^☆^^☆☆^

**DOI:** 10.1016/j.mcn.2013.04.002

**Published:** 2013-09

**Authors:** Miha Modic, Jernej Ule, Christopher R. Sibley

**Affiliations:** a MRC Laboratory of Molecular Biology, Hills Road, Cambridge, CB2 0QH, UK; b Gene Center and Department of Biochemistry, Ludwig-Maximilians-Universität München, Feodor-Lynen-Strasse 25, 81377 Munich, Germany; c Department of Molecular Neuroscience, UCL Institute of Neurology, Queen Square, London WC1N 3BG, UK

**Keywords:** CLIP, Neurodegeneration, FUS, Muscleblind, RNA binding proteins

## Abstract

The fate of an mRNA is largely determined by its interactions with RNA binding proteins (RBPs). Post-transcriptional processing, RNA stability, localisation and translation are some of the events regulated by the plethora of RBPs present within cells. Mutations in various RBPs cause several diseases of the central nervous system, including frontotemporal lobar degeneration, amyotrophic lateral sclerosis and fragile X syndrome. Here we review the studies that integrated UV-induced cross-linked immunoprecipitation (CLIP) with other genome-wide methods to comprehensively characterise the function of diverse RBPs in the brain. We discuss the technical challenges of these studies and review the strategies that can be used to reliably identify the RNAs bound and regulated by an RBP. We conclude by highlighting how CLIP and related techniques have been instrumental in addressing the role of RBPs in neurologic diseases. This article is part of a Special Issue entitled: RNA and splicing regulation in neurodegeneration.

## Introduction

As an mRNA progresses through the various regulatory stages, from transcription to translation and degradation, it is associated with a myriad of multifunctional RNA-binding proteins (RBPs). Indeed, recent studies suggest that hundreds of RBPs interact with mRNAs to collectively determine their fate (Baltz et al., 2012; Castello et al., 2012). For example, many RBPs are components of multi-protein complexes such as the spliceosome or ribosome that mediate specific aspects of gene expression. Others can directly interact with pre-mRNAs, mRNAs and other non-coding RNAs to control their processing, localisation, stability and structure. The importance of RBPs is further highlighted by the fact that aberrant RBP interactions with RNA can lead to several human pathologies, including neurodegenerative diseases, making their study of great importance. Here we briefly discuss what the genome-wide technologies have taught us about post-transcriptional regulation by RBPs that are implicated in neurodegenerative disease.

## Identifying the RNA targets of an RBP

**Fig. 1:**
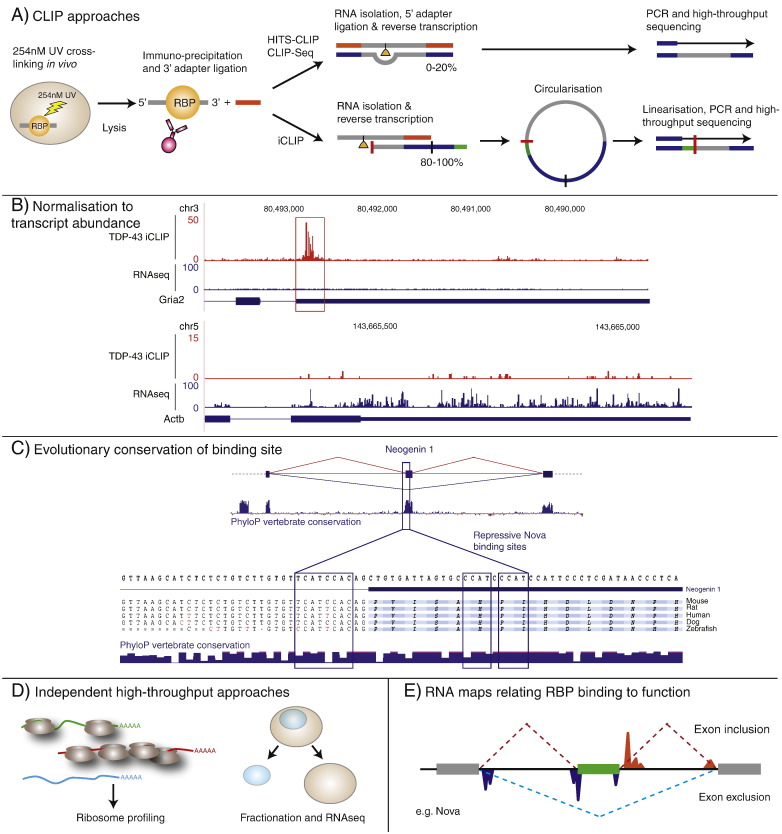
Identifying RNA targets of an RBP with CLIP. A) Schematic representation of HITS-CLIP, CLIP-Seq and iCLIP procedures. Samples are initially cross-linked with 254 nm UV before being lysed and the RBP-of-interest immuno precipitated together with bound RNA. A 3′ adapter is ligated to the RNA and integrity of RBP–RNA complexes examined following isotopic labelling and denaturing electrophoresis (not shown). RNA is released from the RBP by proteinase digestion. In standard CLIP protocol (used in HITS-CLIP, CLIP-Seq and PAR-CLIP), a 5′ adapter is ligated to the released RNA — therefore, only the cDNAs that pass across the cross-link site can be identified. In iCLIP, the second adapter is included as an overhang on the primer used for reverse transcription. Circularisation of cDNAs, followed by linearisation, enables iCLIP to identify the cDNAs truncating at the cross-link site. B) Normalisation to RNAseq reveals enriched clusters of TDP-43 binding to the Gria2 3′UTR above background in the embryonic day 18 mouse brain (red box), and shows that binding to the 3′UTR of the β-actin transcript is an artefact of high RNA abundance. Scales on y-axis represent number of CLIP/RNAseq tags detected. C) Evolutionary conservation of repressive Nova binding sites around an alternative exon of Neogenin 1. D) Confidence in the identification of CLIP targets can be improved when CLIP data is compared to other high-throughput approaches in which the RBP is manipulated. This can include ribosome profiling and knockdown RNAseq analysis of different cell fractions to assess changes in locations. E) CLIP data can be integrated into RNA maps which predict how RBP binding determines mechanistic outcomes on an RNA target.

A crucial challenge of researchers studying the functions of RBPs is to identify the RNAs regulated by a specific RBP. CLIP enables study of RBP–RNA interactions that occur in intact cells or tissues since the biological samples are UV cross-linked, forming a covalent bond between the RNAs in direct contact with the RBP. The RBP-of-interest is then immuno precipitated, and the co-purified RNA is ligated to adapters that enable production of cDNA libraries for sequencing (Ule et al., 2003) (Fig. 1A). CLIP allows unbiased transcriptome-wide analysis of protein–RNA interactions, which is a crucial step towards unravelling the mechanisms of post-transcriptional regulatory networks. However, it is important to appreciate the challenges of CLIP, and the need to integrate it with other technologies.

A common expectation from the CLIP studies is that they will directly generate a shortlist of the functionally relevant RNA targets, enabling the study of pathological relevance of these RNAs and the development of new RNA-based therapies. CLIP is clearly a step towards this goal. However, each RBP can regulate a great multitude of RNAs by diverse mechanisms which presents a greater challenge than appreciated at first sight. The frequency of CLIP sequence reads that identify a specific site depends not only on the affinity of the RBP for this site, but also on the RNA abundance. It is clear that RNA abundance varies between genes, and can vary also within each gene due to variable kinetics of transcription, splicing and intronic RNA degradation. It remains challenging to adequately account for these factors in data analysis. The tendency to report the absolute number of RNA targets should thus be approached with caution, since these numbers depend on the variable quantitative quality of CLIP cDNA libraries and require the use of arbitrary binding thresholds. In appreciation of these points, we describe several approaches that can be used to increase the confidence in assigning functionally relevant RNA targets of RBPs.

### Produce CLIP data with high resolution and quantitative nature

The original CLIP method (Ule et al., 2003), which was used also in HITS-CLIP and CLIP-Seq protocols (Licatalosi et al., 2008; Yeo et al., 2009), had not been optimised for the purpose of high-throughput sequencing. Therefore, when a limited amount of starting material is available, this method may generate cDNA libraries that contain a limited number of unique sequence reads. UV cross-linking employed by all CLIP methods leads to formation of a covalent bond between the RNA and protein. Upon proteinase digestion to release the RNA after immunoprecipitation, a short poly-peptide is left at the cross-link site which subsequently stalls reverse transcription in 80–100% of cDNAs (Sugimoto et al., 2012). The original CLIP method relies on read-through across the deposited polypeptide during reverse transcription into a 5′ adapter, and therefore doesn't identify the majority of cDNAs that truncate at cross-link sites. This loss is avoided by the individual nucleotide resolution CLIP (iCLIP), a method that captures the truncated cDNAs resulting from stalled reverse transcription and thereby enables a more efficient and comprehensive analysis of protein–RNA interactions (Konig et al., 2010) (Fig. 1A). Importantly, the truncation sites in iCLIP enable nucleotide-resolution analysis of cross-link sites.

The challenge to identify functionally relevant RBP targets of an RBP is particularly great for RBPs that bind to pre-mRNAs in the nucleus. Due to the great length of pre-mRNAs, a binding site for an RBP is present in most expressed pre-mRNAs. If sequencing is carried out to sufficient depth, CLIP studies typically identify multiple binding sites of each RBP in most expressed pre-mRNAs. It is therefore crucial that CLIP data are quantitative, in order to identify discrete regions where high cross-linking density corresponds to high-affinity binding. iCLIP was developed to preserve the quantitative information by using primers for reverse transcription that contain a random barcode. Analysis of this random sequence enables computational filtering of high-throughput sequencing data to remove the artefacts caused by variable PCR amplification of different sequences (Sugimoto et al., 2012).

An alternative method to increase the efficiency of CLIP employs photoactivatable ribonucleoside enhanced cross-linking and immuno-precipitation (PAR-CLIP) (Hafner et al., 2010). However, this method has yet to be used in the brain due to challenges facing the required incorporation of synthetic nucleosides in vivo. A recent review on these technologies provides a more detailed discussion on the quality-control steps that can be taken when using these and other high-throughput studies for studying protein–RNA interactions (Konig et al., 2011).

### Produce a rank-ordered list of RNA-abundance normalised data

A crucial step in the analysis of CLIP data is to identify sites where RBPs bind with high-affinity, which can be identified by clusters of reads (in CLIP) or clusters of cross-link events (in iCLIP or PAR-CLIP). This in turn acts to reduce the prediction of false positive RNA targets. The most common approach used in the past studies is to calculate the probability that a cluster of reads that is present in a specific genomic region (such as an intron or a gene) is statistically significant when real CLIP data are compared to randomised CLIP data within the same region (Konig et al., 2010; Yeo et al., 2009). This approach is particularly appropriate for studying intronic binding events, since intronic RNA abundance has rarely been comprehensively quantified with current high-throughput sequencing methods. One way to approximately measure intronic abundance is to use the average intronic read density of CLIP data.

To normalise mRNA abundance for RBPs that bind to mRNAs, mRNA sequencing (RNAseq) should be carried out simultaneously with CLIP experiments, and used to normalise the number of CLIP reads by the RNAseq reads (Kishore et al. 2011) (Fig. 1B). The primary objective of this approach is to filter the regions with enriched CLIP reads from those which have widespread CLIP reads due to high RNA abundance. This normalisation is particularly important when CLIPing from heterogenous populations of cells in the brain, where expression of mRNA targets can vary greatly, and often the mRNAs with low expression have important cell-type specific functions. The CLIP/RNAseq ratio can then be used to produce a rank-ordered list of RNAs (Darnell et al., 2011; Konig et al., 2010; Polymenidou et al., 2011). Past studies have defined groups of high, medium and low confidence targets for further analyses (Darnell et al., 2011; Konig et al., 2010; Polymenidou et al., 2011). An alternative approach would be to avoid the arbitrary thresholds, and instead use rank-ordered lists as in the Sylamer method, which detects microRNA target sites from a rank-ordered list of genes (van Dongen et al., 2008). A similar approach has recently been used to evaluate the likely mRNA targets of FMRP (Ascano et al., 2012).

### Use comparative genomics to identify the conserved binding sites

Evolutionary conservation of the sequence motifs bound by the protein can support the functional importance of the RNA binding site (Fig. 1C). For instance, analysis of the RNA motifs that confer Nova binding around its regulated exons demonstrated their high conservation between vertebrate species in CLIP identified targets (Jelen et al., 2007). It was found that the most highly conserved Nova binding sites were in genes with indispensable functions in synaptic development. Nevertheless, it must be considered that the analysis of conserved sites will bias against the sites that are functional and contribute to phenotypic diversity within and between species.

### Use independent functional methods

Genome-wide studies have revealed that RBPs often control diverse aspects of post-transcriptional regulation. Initial biochemical studies were based on a small number of regulated RNAs, therefore RBPs that regulate splicing were often classified as splicing repressors or enhancers. Studies of Nova showed that it can enhance or repress exon inclusion in many different RNA targets (Ule et al., 2005), and this has now been seen also for most other RBPs within the subset that are implicated in splicing regulation (Witten and Ule, 2011). Moreover, Nova governs the usage of alternative polyadenylation sites (Licatalosi et al., 2008), and can also control mRNA localisation (Racca et al., 2010). Similarly, a comprehensive functional evaluation of Mbnl1 demonstrated its functions in pre-mRNA processing, translation and control of RNA localisation (Wang et al., 2012). Specifically it was found that Mbnl1 bound transcripts showed a change in ribosomal footprints and redistribution from the membrane fraction to the insoluble fraction upon Mbnl1/Mbnl2 knockdown. Due to this multifunctionality of RBPs, the full functional validation of CLIP datasets will remain a challenge in the years to come. Integration of CLIP with multiple genome-wide methods including RNAseq, ribosom profiling (Ingolia et al., 2011), mRNA localisation studies (Wang et al., 2012) and others will be required to determine the full set of functionally relevant RNA targets of an RBP (Fig. 1D).

### Define position-dependent regulatory principles, or “RNA maps”

Integration of CLIP data with analysis of global changes of splicing has been used to identify the global positional principles governing the splicing functions of Nova (Licatalosi et al., 2008; Ule et al., 2006). The combined findings excitingly identified a position-dependent regulation of these transcripts by Nova proteins and were used to define an RNA map in which exon exclusion is facilitated by binding close to branch points, splice sites or within exons, and exon inclusion favoured by Nova binding downstream of exons (Fig. 1E). Notably, binding sites close to the flanking exons also mediated predictable outcomes (Ule et al., 2006). Among others, this approach has now been used for TDP-43/TARDBP (herein referred to as TDP-43) (Polymenidou et al., 2011; Tollervey et al., 2011a), TIA1/TIAL1 (Wang et al., 2010), FUS/TLS (herein referred to as FUS) (Lagier-Tourenne et al., 2012; Rogelj et al., 2012), FOX2 (Yeo et al., 2009), PTBP1 (Xue et al., 2009) and Mbnl1 (Wang et al., 2012), and has shown that many RBPs have common positional principles in the regulation of splicing. Moreover, an RNA map also underlined the position-dependent activity of Nova proteins in alternative polyadenylation (Licatalosi et al., 2008).

The integrative analysis has three main advantages. Firstly, the RNA map can validate the high quality of data by demonstrating that binding position correlates with the effect of the protein (enhanced vs. repressed), and that binding at these positions in the regulated RNAs is significantly enriched relative to the control RNAs. Secondly, since the RNA map defines the positions where RBPs bind to have functional impact, it enables separation of those changes observed in RNAseq data that are the result of direct effects of the RBP from those that may result from indirect effects. This is particularly important for studies in the brain, since manipulation of RBPs can lead to developmental defects, cell stress, changes in neuronal networks or glia–neuron interactions, siRNA off-targets, and other effects that can indirectly change gene expression. Thirdly, the positional pattern in the RNA map can reveal new regulatory mechanisms employed by the RBPs. For instance, RNA maps demonstrated that the enhancer elements are most often located immediately downstream of exons. Moreover, they showed that certain RBPs can also regulate splicing via distal binding sites (Witten and Ule, 2011).

## CLIPing the brain in health and disease

To date, RNA binding profiles of several RBPs involved in neurologic disorders have been investigated either in cell culture, transgenic animal models, or human post-mortem tissue (Table 1). Here we choose representative RBPs to demonstrate how CLIP studies helped understand their activity in the CNS, and thereby provide the foundation for understanding their role in disease pathologies.

### Nova

Paraneoplastic opsoclonus myoclonus ataxia is a neurological disorder mediated by autoimmune attack against onconeural disease antigens including Nova-1 (Buckanovich et al., 1993) and Nova-2 (Yang et al., 1998), and was the study of initial CLIP experiments. Low-throughput (Ule et al., 2003) and, more recently, high-throughput (Licatalosi et al., 2008) sequencing of Nova CLIP libraries revealed the RNA map in which exon exclusion is facilitated by binding close to branch points, splice sites or within exon, and exon inclusion favoured by binding downstream of exons (Ule et al., 2006). Activity is specifically directed by binding to YCAY clusters within target genes, with nuclear-cytoplasmic shuttling of certain targets possible due to Nova's nuclear localisation and nuclear export sequences (Racca et al., 2010). This has been followed up by showing that Nova can act outside of splicing to regulate alternative polyadenylation through binding of clusters flanking poly-A sites, although the relevance to disease remains unknown (Licatalosi et al., 2008). By identifying the mRNAs that are directly regulated by Nova proteins (Licatalosi et al., 2008; Ule et al., 2006), the Nova RNA map enabled studies that revealed how specific mRNAs mediate the function of Nova proteins in the brain. In particular, defective splicing of *Agrn* and *Dab1* leads to defects in the formation of the neuromuscular junction and in neuronal migration in the CNS of the Nova1^−/−^/Nova2^−/−^ mice, respectively (Ruggiu et al., 2009; Yano et al., 2010).

### FMRP

Loss of function of fragile-X mental retardation protein (FMRP) is responsible for fragile-X syndrome, the most common inherited form of mental retardation. FMRP is an important regulator of translation (Khandjian et al., 2004; Laggerbauer et al., 2001; Li et al., 2001). A study using CLIP in mouse brain reported that FMRP binds the coding sequence of mRNAs with little sequence specificity. By uncovering FMRP interacting targets in polyribosomal enriched samples, it was shown that FMRP binding is enriched on open reading frames of a collection of mRNAs encoding synaptic transcripts, including multiple receptor complexes and G-protein signalling pathways (Darnell et al., 2011). A recent study using FMRP PAR-CLIP in HEK293 cells additionally reported two binding motifs recognised by FMRP: ACUK and WGGA (in which K = G or U and W = A or U). An integration of PAR-CLIP data with motif analysis was used to determine a ranked enrichment of FMRP mRNA targets. Interestingly, 93 of the top ranked genes are implicated in autism spectrum disorders (Angelman, Prader–Willi, Rett, and Cornelia de Lange syndromes). FMRP was found to regulate protein levels of several of these genes in human cell culture, mouse ovaries and human brain (Ascano et al., 2012). Interestingly, a recent study of CELF4, another neuronal RBP, showed that it shares more than 30% of its RNA targets with FMRP, and is even more strongly enriched in transcripts linked to autism spectrum disorders (Wagnon et al., 2012).

### Mbnl

Muscleblind-like proteins (Mbnl1 and Mbnl2) are sequestered in various tissues within the neuromuscular diseases of myotonic dystrophy types 1 (DM1) and 2 (DM2) in response to expansions of CUG repeats in the 3′UTR of the myotonic dystrophy protein kinase (*DMPK*) (Harley et al., 1992; Mahadevan et al., 1992), or CCUG repeats in the zinc finger protein 9 (*ZNF9*) (Ranum et al., 1998), respectively. Previously known to be regulators of splicing, two groups have now integrated CLIP, RNAseq and ribosome profiling to confirm that nuclear Mbnl proteins bind introns, coding sequences and 3′UTRs at UGC or GCU-containing 4-mers to dictate splicing changes and identify direct targets for splicing regulation based on CLIP-identified binding (Charizanis et al., 2012; Wang et al., 2012). The RNA map reveals that Mbnl proteins bind in upstream introns and alternative exons to promote exclusion, and in downstream introns to promote inclusion of exons. Further, a previously described role of Mbnl proteins in regulating localisation in the cytoplasm (Adereth et al., 2005) was extended transcriptome-wide, whilst the aforementioned regulation of the translation state of 3′UTRs carrying Mbnl1 binding sites provides further insight into disease mechanisms when this protein is sequestered (Wang et al., 2012). It will now be important to assess if these perturbations in patient samples with CUG repeats correlate with disease severity.

### FUS and TDP-43

ALS and FTLD are two diseases with phenotypic and mechanistic overlap (Ferrari et al., 2011; Lagier-Tourenne and Cleveland, 2009). Familial mutations to TDP-43 and FUS have been identified in each disease (Broustal et al., 2010; Kabashi et al., 2008; Kwiatkowski et al., 2009; Van Langenhove et al., 2010; Vance et al., 2009), and these proteins aggregate abnormally in neurons during disease progression. Despite these common links, CLIP studies of TDP-43 and FUS did not find a significant overlap in their RNA binding sites. Whereas TDP-43 has a strong specificity to bind distinct UG-rich RNA regions (Polymenidou et al., 2011; Tollervey et al., 2011a), FUS binds with mild specificity to G-rich motifs along the full-length of pre-mRNA transcripts (Ishigaki et al., 2012; Lagier-Tourenne et al., 2012; Rogelj et al., 2012).

Another observation made by FUS CLIP was a saw-tooth binding pattern along long introns, similar to that seen in total RNAseq (Ameur et al., 2011; Lagier-Tourenne et al., 2012; Rogelj et al., 2012). However, whether this binding profile is distinct and disease-relevant remains a matter of debate. This pattern is readily observed for FUS in long introns (Ameur et al., 2011; Lagier-Tourenne et al., 2012), but can also be observed in shorter introns using the increased coverage and quantitative ability of the iCLIP method (Rogelj et al., 2012). Although suggested to be unique to FUS (Lagier-Tourenne et al., 2012), iCLIP analysis showed that TDP-43, U2AF65 and FUS all had a linear increase in crosslinking as the distance from the 3′ splice site increases, similar to the increase in total RNAseq (Rogelj et al., 2012). This suggests that the saw-tooth pattern may be an indirect result of the variable abundance of the intronic RNA, which increases linearly with the distance from the 3′ splice site.

Interestingly, FUS and TDP-43 regulate splicing of different alternative exons (Lagier-Tourenne et al., 2012; Rogelj et al., 2012). However, analysis of RNAseq data showed an overlap in the long genes, which were sensitive to loss of function of FUS and TDP-43 in transgenic models and in neurons derived from patients with familial mutations to FUS (Lagier-Tourenne et al., 2012; Polymenidou et al., 2011). It will be important to understand the mechanisms behind changes in expression of long genes, and their role in disease pathology. Whereas the effect on long genes may involve novel transcriptional functions of TDP-43 and FUS, it could also result from accumulated defects in pre-mRNA processing. RBPs can promote stability of mRNAs by preventing recognition of cryptic exons; for example, knockdown of *hnRNPC* leads to widespread expression of cryptic exons, which can result in decreased gene expression. In the absence of hnRNP C, cryptic exons are recognised by U2AF65, a splicing factor that initiates the recognition of 3′ splice sites (Zarnack et al., 2012). RNA splicing maps demonstrate that similar to hnRNP C, FUS and TDP-43 bind close to 3′ splice sites to repress splicing, where they might displace U2AF65 (Rogelj et al., 2012; Tollervey et al., 2011a). Due to the saw-tooth pattern, U2AF65 has increased binding in long introns (Rogelj et al., 2012), which could increase the danger of recognising cryptic exons, and thereby decreasing expression of long genes. Aberrant binding of U2AF65 could be caused by loss of other RBPs that regulate splicing in the brain. Therefore, it will be important to study gene expression in transgenic models of other RBPs to assess if the effect on long genes is specific to TDP-43 and FUS.

## Future considerations for CLIP studies in the CNS

CLIP and its variants are now the primary method for studying protein–RNA interactions and we have described how several early high-throughput studies have provided invaluable insights into the mechanisms of neurodegenerative diseases. This includes the identification of putative RNA targets and understanding the way in which an RBP acts through looking at its specificity and the regions bound within a transcript. It is likely that CLIP-based approaches will remain popular, in part due to recent method developments making it a more robust, and also following the recent identification of many new candidate RBPs using the global crosslinking-based purification (Baltz et al., 2012; Castello et al., 2012).

We explain the limitations in reporting a precise number of RNA targets of an RBP based on CLIP. CLIP tags alone are not sufficient to determine the functionally relevant binding sites of RBPs. Therefore it is important to integrate CLIP with other approaches in order to identify the RNAs that are bound and directly regulated by the RBPs. For RBPs interacting with mRNAs we encourage the use of normalisation of CLIP clusters to transcript abundance either using RNAseq, whereas other approaches are necessary for CLIP data normalisation of RBPs binding pre-mRNAs. Evaluation of evolutionary conservation, integration of complementary high-throughput methods, and generation of RNA maps can help to unify the identification of functionally relevant RNA targets for subsequent studies.

One application of CLIP that remains to be widely applied is the study of protein–RNA interactions in human postmortem tissues (Tollervey et al., 2011a). It is important to appreciate that cell stress and neuronal loss can obscure the causative mechanisms in postmortem tissues. This has become particularly apparent from the study of gene expression of FTLD and Alzheimer disease temporal cortex, which found widespread yet identical changes in gene expression (Tollervey et al., 2011b). Whilst CLIP techniques are compatible with partially degraded postmortem tissue, it is important to appreciate that there are many variables in sample preparation that can affect the quality of the sample. This can include the length to autopsy and sample collection, as well as the fixation and freezing methods employed. Therefore special care needs to be taken when analysing data produced from samples collected under variable conditions.

The studies of postmortem tissues are also inevitably correlative, since it is not possible to go back to the human tissue to manipulate the function of the RBP. Therefore, it is important to additionally study the relevant protein–RNA interactions in disease model systems. Transgenic animal models have been used most often but a new opportunity is arising in the study of pluripotent stem cell (PSC) disease models (Patani et al., 2012). Induced PSCs (iPSCs) of TDP-43 M337V mutation have now been generated which have elevated levels of TDP-43 protein and decreased survival following neuronal differentiation (Bilican et al., 2012), whilst FUS has already been knocked down in differentiated neural stem cells and displayed comparative changes to both transgenic models and patient tissue (Lagier-Tourenne et al., 2012). If disease phenotype continues to be recapitulated for other disease-associated proteins, then iPSCs will represent an invaluable sample source for future CLIP studies.

Over the next years, we can expect more CLIP studies to assess RNA interactions of multiple RBPs at a specific stage of brain development, which will enable comprehensive comparative studies. It will be possible at that point to define the phenomena specific to RBPs involved in neurodegenerative disorders, and separate these from the more general aspects of protein–RNA interactions.

## Figures and Tables

**Table 1:** CLIP studies on RBPs implicated in neurological function in health and disease. Abbreviations: NOVA1/2 — neuro-oncological ventral antigen 1/2, TARDP — TAR DNA binding protein, FMRP — fragile-X mental retardation protein, MBNL1/2 — muscleblind-like protein 1/2, FUS/TLS — fused in sarcoma/translocated in liposarcoma, PTBP2 — polypyrimidine tract binding protein 2, PARK7 — Parkinson protein 7, ELAVL1 — embryonic lethal, abnormal vision, Drosophila-like 1, CELF4 — CUGBP Elav-like family member 4.

Symbol	Disease	Key findings	Reference
CELF4	Epilepsy	Binds UGU motifs in 3′UTRs.	Wagnon et al. (2012)
Hyperactivity	Controls stability of mRNAs encoding synaptic proteins.	
ELAVL1	Epilepsy	Recognises U-rich stretches interspersed with Gs.	Ince-Dunn et al. (2012)
Regulates transcript stability.	
Controls the synthesis of glutamate.	
FMR1	Fragile-X mental retardation	Represses the translation of target mRNAs.	Darnell et al. (2011)
Autism spectrum disorders	Preferred binding to the coding region of exons.	Ascano et al. (2012)
Increased association with transcripts encoding synaptic proteins.	
Binds ACUK and WGGA (in which K = G or U and W = A or U) motifs.	
FUS	Frontotemporal lobar degeneration, amyotrophic lateral sclerosis	Binds along the full length of pre-mRNAs.	Ishigaki et al. (2012)
Regulates alternative splicing of many neuronal development genes.	Rogelj et al. (2012)
Knockdown leads to decreased expression of long genes in the brain.	Lagier-Tourenne et al. (2012)
MBNL1/2	Myotonic dystrophy (DM)	Recognises UGC or GCU-containing 4-mer clusters.	Wang et al. (2012)
Regulates DM-related alternative splicing.	Charizanis et al. (2012)
Contributes to mRNA localisation and translation by binding to 3′UTRs.	
NOVA1/2	Paraneoplastic opsoclonus-myoclonus-ataxia (POMA)	Binds YCAY clusters to regulate alternative splicing.	Licatalosi et al. (2008)
Controls synaptogenesis and neuronal migration via specific mRNAs.	Ruggiu et al. (2009)
Regulates alternative poly-adenylation in the brain.	Yano et al. (2010)
PARK7	Parkinson's disease	Recognises CC/GG rich regions.	van der Brug et al. (2008)
Inhibits translation of target mRNAs.	
PTBP2		Recognises UCU-rich motifs to regulate alternative splicing.	Licatalosi et al. (2012)
Regulates neural stem cell polarity in developing brain.	
Involved in mRNA trafficking stability and translation.	
TARDBP	Frontotemporal lobar degeneration, amyotrophic lateral sclerosis	Recognises UG repeats and UG-rich motifs in introns and 3′ UTRs.	Tollervey et al. (2011a)
Regulates alternative splicing of many neuronal development genes.	Polymenidou et al. (2011)
Knockdown leads to decreased expression of long genes in the brain.	
